# Decitabine improves MMS-induced retinal photoreceptor cell damage by targeting DNMT3A and DNMT3B

**DOI:** 10.3389/fnmol.2022.1057365

**Published:** 2023-01-10

**Authors:** Yanli Ji, Meng Zhao, Xiaomeng Qiao, Guang-Hua Peng

**Affiliations:** ^1^Laboratory of Visual Cell Differentiation and Regulation, Basic Medical College, Zhengzhou University, Zhengzhou, China; ^2^Department of Pathophysiology, Basic Medical College, Zhengzhou University, Zhengzhou, China; ^3^Department of Forensic Medicine, Basic Medical College, Zhengzhou University, Zhengzhou, China

**Keywords:** retinitis pigmentosa, DNA methylation, DNMT inhibitor, methyl methanesulfonate, DNA damage response

## Abstract

**Introduction:**

Retinitis pigmentosa (RP) is a group of neurodegenerative retinopathies causing blindness due to progressive and irreversible photoreceptor cell death. The alkylating agent methyl methanesulfonate (MMS) can induce selective photoreceptor cell death, which is used to establish RP animal models. MMS induces DNA base damage by adding alkyl groups to DNA, and epigenetic modifications influence DNA damage response. Here, we aimed to explore the relationship between DNA methylation and DNA damage response in dying photoreceptors of RP.

**Methods:**

The mouse RP model was established by a single intraperitoneal injection of MMS. The retinal structure and function were assessed by H&E, OCT, TUNEL, and ERG at several time points. The expression of DNA methylation regulators was assessed by qPCR and Western blot. DNMT inhibitor 5-aza-dC was applied to inhibit the activity of DNA methyltransferases and improve the retinal photoreceptor damage.

**Results:**

The outer nuclear layer (ONL) and IS/OS layer were significantly thinner and the retinal function was impaired after MMS treatment. The cell death was mainly located in the ONL. The retinal damage induced by MMS was accompanied by hyperexpression of DNMT3A/3B. The application of DNMT inhibitor 5-aza-dC could suppress the expression level of DNMT3A/3B, resulting in the remission of MMS-induced photoreceptor cell damage. The ONL and IS/OS layers were thicker than that of the control group, and the retinal function was partially restored. This protective effect of 5-aza-dC was associated with the down-regulated expression of DNMT3A/3B.

**Conclusion:**

These findings identified a functional role of DNMT3A/3B in MMS-induced photoreceptor cell damage and provided novel evidence to support DNMTs as potential therapeutic targets in retinal degenerative diseases.

**Graphical Abstract:**
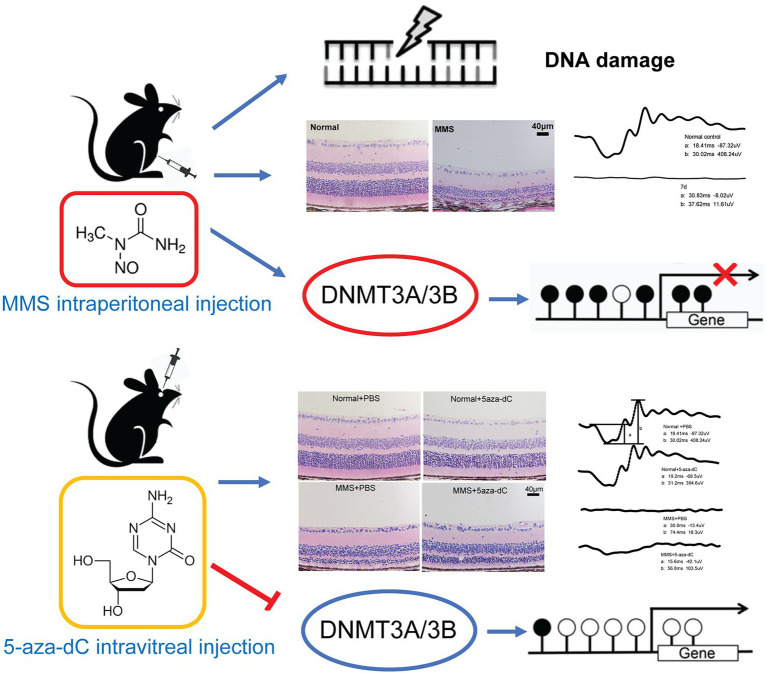

## 1. Introduction

Retinitis pigmentosa (RP) is a group of neurodegenerative retinopathies causing blindness due to progressive and irreversible photoreceptor cell death, afflicting about 15 million patients all over the world (Olivares-Gonzalez et al., 2021). The main clinical characteristics of RP include adolescent night blindness, progressive loss of the peripheral visual field, retinal pigmentation, and abnormal electroretinogram (Hartong et al., 2006). Nowadays, several therapeutic strategies, such as gene editing (Wang et al., 2019), stem cell or retinal progenitor cell transplantation, implant-prosthetic therapy and neuroprotection, are under study. However, only a few of them have been carried out into the clinical stage.

The alkylating agents widely exist in the natural environment, which could induce DNA base damage by adding alkyl groups to DNA (3MeA, 7MeG, and O^6^MeG) resulting in cell death (Meira et al., 2009). Methyl methanesulfonate (MMS) and methyl nitrosourea (MNU) are two main kinds of alkylating agents. These agents can selectively induce photoreceptor cell death without affecting other retinal neurons (Meira et al., 2009; Allocca et al., 2019). Therefore, they are widely used to establish retinal degenerative animal models (Reisenhofer et al., 2015; Calvo et al., 2016; Allocca et al., 2017; Tao et al., 2019b). In the previous studies, both apoptotic and necrotic cell death pathways were observed in MMS-induced retinal degenerative mice models at 2 or 3 days post-injection (Meira et al., 2009; Allocca et al., 2019). In addition, parthanatos which is a process dependent on PARP1 hyperactivation was also detected in the MMS model (Calvo et al., 2013; Allocca et al., 2019). Studies showed alkylating agents involved in many cellular pathways that induced DNA damage responses such as direct reversal, base excision repair (BER), and mismatch repair (MMR; Sedgwick et al., 2007). Individual responses varied significantly, which implied that both genetic and epigenetic mechanisms participated in modulating the alkylating agent toxicity (Fu et al., 2012). MMS is a methylating agent, which directly introduces the methyl group to DNA to methylate the O-6 atom of guanine (Araujo-Lima et al., 2018). DNA methylation can act through covalent modification of DNA, generating mismatched base derivatives and lesions which interrupt genetic replication (Jiang et al., 1999). It has not been reported whether MMS stimulates the remodeling of epigenetic modifications during this process and whether the methylation of guanine can activate DNA methyltransferases.

DNA methylation is a universal and well-studied epigenetic mechanism, maintaining dynamic balance by DNA methyltransferases (DNMTs) and demethylases (TETs) throughout the whole life (Skvortsova et al., 2019). Many physiological and pathological processes, such as aging, cancer, nutritional situation changes, and environmental exposures, are related to DNA methylation. Studies in several RP animal models (rd1, rd2 mice and P23H, S334ter rats) showed that DNA hypermethylation was detected in dying photoreceptors and accompanied by higher expression of *Dnmt3a* (Wahlin et al., 2013; Farinelli et al., 2014). DNMT inhibitor decitabine reduced the DNA methylation level and the number of TUNEL-positive cells in the short-term investigation (Farinelli et al., 2014). Knockdown of *Dnmt3b* led to abnormal retinal pigment epithelial cells (RPE) and disorganized retinal laminations (Nasonkin et al., 2011). The expression levels of DNMT3A/3B and MBD4 (Methyl-CpG binding domain protein) were increased and the MBD2 was reduced in age-related macular degeneration (AMD; Nashine et al., 2019). Moreover, DNA methylation also influences chemical agent-induced DNA damage response. DNMT inhibitors reduced tert-butyl hydroperoxide-induced DNA damage in the retinal pigment epithelial cells by increasing the expression of antioxidant genes (Tokarz et al., 2016). Thus, DNA methylation has been implicated in the pathogenesis of retinal damage. However, it is unclear whether DNA methylation is involved in the process of alkylation agents-induced photoreceptor cell death, if so, how it changes, and whether DNMT inhibitors might able to prevent retinal damage.

Hence, we investigated the relationship between DNA methylation and MMS-induced specific photoreceptor cell injury. The RP model was established by a single intraperitoneal injection of MMS (75 mg/kg body weight) in mice. The time course of retinal morphological and functional changes was monitored. The TdT-mediated dUTP nick-end labeling (TUNEL) assay was used to mark the dying neurons. The expression levels of DNA methylation regulators were detected *via* quantitative real-time RT–PCR analysis and western blot. Finally, the role of methyltransferase inhibitor decitabine in MMS-induced retinal injury was determined. This study will provide a new insight and theoretical basis for understanding the epigenetic mechanisms of chemical-induced retinal injury.

## 2. Materials and methods

### 2.1. Animals and MMS treatment

Wild-type adult male C57BL/6J mice (7–8-week-old) were purchased from Vital River Laboratories (Beijing, China). All animal experiments were performed by the ARVO Statement for the Use of Animals in Ophthalmic and Vision Research and were approved by the Institutional Animal Care and Use Committee of Zhengzhou University. All mice were housed under standard conditions (12/12-h light/dark cycle, 40–60% humidity, room temperature 18–23°C), with water and food available *ad libitum*. For the MMS treatment experiment, mice were randomly divided into two groups: Normal control group (*n* = 6), MMS groups (*n* = 6/time point). The treated mice have received a single intraperitoneal injection of MMS (75 mg/kg, Sigma-Aldrich, United States) dissolved in PBS, and the control mice were left without any drug administration. Mice were examined at several different time points (4, 12, 18 h, 1, 2, 3, 5, and 7 days post-injection).

### 2.2. 5-aza-dC intravitreal injection

Experimental mice were randomly divided into two groups: Normal control (*n* = 12) and MMS (*n* = 12). All mice were anesthetized by intraperitoneal injection of pentobarbital (50 mg/kg body weight) and given proparacaine hydrochloride eye drops (Alcon Laboratories, Fort Worth, TX, United States) for topical anesthesia. 1 μl of 5-aza-dC (2 μM, Decitabine, Sigma) dissolved in PBS was administered by intravitreal injection in the right eye, and 1 μl vehicle (PBS) in the left eye, 24 h before MMS administration. Eyes were enucleated at 7 days post injury after the *in vivo* ERG examination, and prepared for HE and western blot analysis.

### 2.3. Full-field electroretinogram recording

Mice were dark-adapted for at least 2 h in a darkroom before the examination. During the procedures of ERG recordings, only a dim red light was on to preserve dark adaptation. Mice were inhaled with 2% isoflurane for initial induction and 1% for maintaining anesthesia mixed with 0.2 L/min of air (Isoflurane Vaporizer, China). Once the animals were sedated with smooth and steady breathing, tropicamide phenylephrine eye drops (Santen Pharmaceutical Co., Ltd. Japan) were applied for pupil dilation and proparacaine hydrochloride eye drops (Alcon Laboratories, Fort Worth, TX, United States) for topical anesthesia. Mice were then transferred to the ERG apparatus (RETI-scan21, Roland Consult, Germany). The corneal gold ring electrodes were placed on the surface of both corneas as the recording electrodes. The needle electrodes, as the ground and reference electrodes, were inserted subcutaneously near the tail and both sides of the cheek, respectively. Artificial tear eye drops were applied on both corneas to keep moisture. Full-field electroretinogram (ffERG) examinations were performed according to the International Society for Clinical Electrophysiology of Vision (ISCEV) standard protocols with two or four light stimuli per recording (McCulloch et al., 2015). The scotopic 3.0 ERG represents maximal combined responses arising from photoreceptors and bipolar cells, mainly from the rod system (Johnson et al., 2019). The b-wave amplitudes were extracted from the dark-3.0 ERG recordings for quantification and analysis statistically.

### 2.4. Optical coherence tomography

*In vivo* retinal structures were assessed using spectral domain-optical coherence tomography (OCT) (Envisu-R2200, Leica Microsystems, United States). Mice were sedated by intraperitoneal injection of 5% chloral hydrate. Pupils were dilated using tropicamide phenylephrine eye drops. The retinal volumetric images centered on the optic nerve head (ONH) were acquired using SD-OCT with parameters of 1.4 mm × 1.4 mm at 1000 A-scan × 100 B-scan × 5. The total retinal thickness from the RNFL to RPE was measured using the software (InVivoVue Diver 2.5) 4 points per image.

### 2.5. Hematoxylin and eosin (H&E) staining

After the *in vivo* examinations, we enucleated the eyes at the given time points, and the enucleated eyes were immersed in the eye-specific fixative cocktail (supplied by Dr. Shaojun Wang) at 4°C no less than 24 h. After complete fixation, the anterior segments of the eyeballs were removed and the eyecups were dehydrated in a graded series of ethanol and embedded in paraffin wax. Vertical sections of the eyecup through the optic nerve were made at 4 μm and stained with H&E. All slides were examined using light microscopy (BX53, Olympus, Japan). The thickness of the ONL and/or IS/OS layer was measured using ImageJ software (National Institutes of Health, Bethesda, MD, United States).

### 2.6. TdT-mediated dUTP nick-end labeling (TUNEL) assay

TUNEL assay was performed with a One-Step TUNEL Apoptosis Assay kit (Beyotime, China). According to the protocols, deparaffinized retinal sections were permeabilized by proteinase K (20 μg/ml) diluted in 10 mM Tris-HCl (pH7.4) at room temperature for 30 min and then washed with 1 × PBS thoroughly. Sections were incubated with TUNEL reaction mixture under a dark and humidified atmosphere for 1 h at 37°C. For the nuclei staining, DAPI was applied to sections for 3 min at room temperature. Images were taken using a fluorescence microscope (BX53, Olympus, Japan). TUNEL-positive cells and photoreceptor cells were counted using ImageJ software and cell death index (CDI) was calculated based on the number of TUNEL-positive cells and total photoreceptor cells (DAPI marked).

### 2.7. Quantitative real-time RT-PCR analysis

According to the manufacturer’s instructions, retinal total RNA was extracted using RNAeasy™ micro kit (Beyotime, China). The complementary DNA was synthesized using the RevertAid First Strand cDNA Synthesis Kit (Thermo Fisher Scientific, United States) and subjected to quantitative real-time PCR (QuantStudio 5, Applied Biosystems, United States) using PowerUp SYBR Green Master Mix (Life Technologies, United States). Primers used in qRT-PCR were shown in Table 1. Relative mRNA levels were determined by the 2^−∆∆CT^ method. *Gapdh* was used as a housekeeping gene.

**Table 1:** Primers of mouse *Dnmt1, Dnmt3a, Dnmt3b, Mecp2, Tet1, Tet2* and *Tet3*.

GeneSymbol	Sequence
*Dnmt1*	F: 5′-GGACAAGGAGAATGCCATGAAGC-3′ R: 5′-TTACTCCGTCCAGTGCCACCAA-3′
*Dnmt3a*	F: 5′-CGCAAAGCCATCTACGAAGTCC-3′ R: 5′-GCTTGTTCTGCACTTCCACAGC-3′
*Dnmt3b*	F: 5′-CGCACAACCAATGACTCTGCTG-3′ R: 5′-GGTGACTTCAGAAGCCATCCGT-3′
*Mecp2*	F: 5′-CGTGACCGGGGACCTATGTAT-3′ R: 5′-CCTCTCCCAGTTACCGTGAAG-3′
*Tet1*	F: 5′-GAGAGATTTCTCGGGTCAGCA T-3′ R: 5′-TTCCTCCTCTCCACCATTGG-3′
*Tet2*	F: 5′-TGTTGTTGTCAGGGTGAGAATC-3′ R: 5′-TCTTGCTTCTGGCAAACTTACA-3′
*Tet3*	F: 5′-CCGGATTGAGAAGGTCATCTAC-3′ R: 5′-AAGATAACAATCACGGCGTTCT-3′
*Gapdh*	F: 5′-TGACCTCAACTACATGGTCTACA-3′ R: 5′-CTTCCCATTCTCGGCCTTG-3′

### 2.8. Western blotting analysis

To assess the protein expression levels of DNA methylation associated factors in the retina, mice were euthanized and retinas were isolated immediately and homogenized in RIPA lysis buffer with protease inhibitors (1% phosphatase inhibitor cocktail and 1 mM PMSF). Then centrifuged and collected supernatants were subject to western blotting analysis. Proteins were loaded in SDS-PAGE gels (10%, 10 μg per lane) and then transferred to PVDF membrane (Millipore, United States). The membrane was blocked with 5% non-fat dry milk in Tris-buffered saline with 0.1% Tween-20 (TBS-T, pH = 7.4) for 2 h at room temperature and incubated overnight with primary antibodies against DNMT1/3A/3B (Epigentek, United States), MeCP2 (Abcam, United States), and GAPDH (Proteintech, United States) at 4°C. Then the membrane was incubated with the appropriate peroxidase-linked secondary antibodies (Proteintech, United States) for 2 h at room temperature. Immunoblots were examined using an ECL detection reagent (Epizyme Biotech, China) and images were captured and analyzed with ChemiDoc Touch Imaging System with Image Lab Touch Software (BioRad, United States).

### 2.9. Statistical analysis

All experiments had more than three biological replicates and all data were expressed as the mean ± SD. Statistical analyses were performed using GraphPad Prism 8.0 software. When different time points groups were compared, a one-way analysis of variance (ANOVA) was performed, followed by Tukey’s test for *post-hoc* comparisons. For MMS and 5-aza-dC treatment comparisons, two-way ANOVA with sidak’s *post-hoc* test was performed. *p* < 0.05 was considered to be statistically significant.

## 3. Results

### 3.1. MMS induces mouse photoreceptor cell specific damage with time

**Figure 1:**
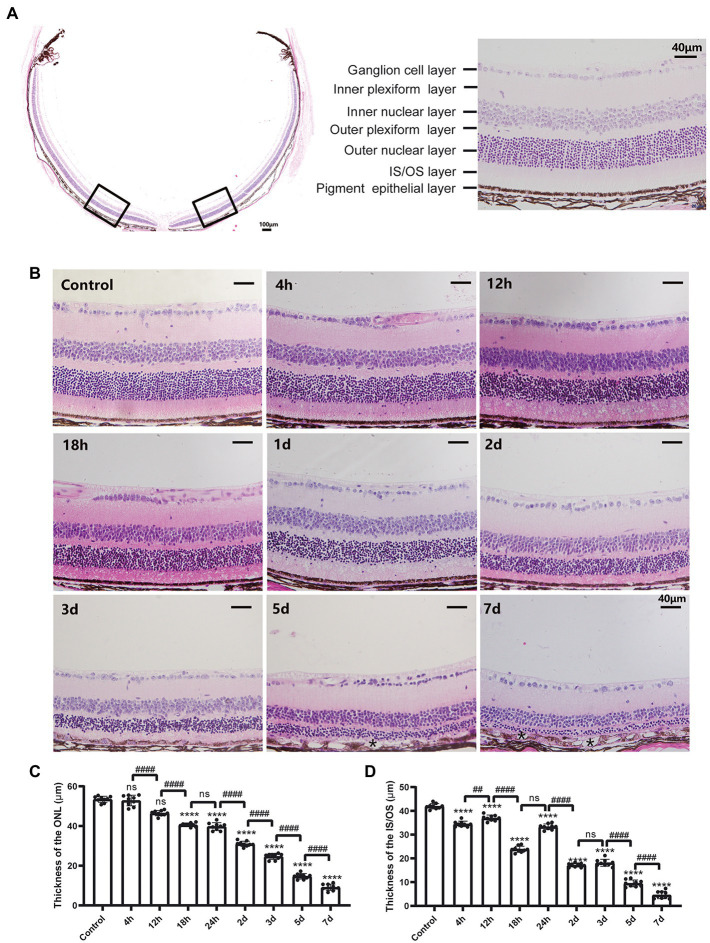
Morphological changes of the retina after MMS treatment with time. **(A)** The representative image of the whole cross-section profile of normal mouse retina (20×, scale bar, 100 μm; 40×, scale bar = 40 μm). **(B)** Retinal morphological changes of different MMS treatment time points. Both of the ONL and IS/OS layers were thinner with time. At 5 and 7 days post MMS injection, * indicates the RPE depigmentation and vacuoles formation (40×, scale bar = 40 μm). **(C)** The thickness of the ONL after MMS treatment with time. **(D)** The thickness of the IS/OS after MMS treatment with time. Data were expressed as mean ± SD, *n* = 3/group. One-way ANOVA followed by Tukey’s *post hoc* test, ****p < 0.0001 for differences compared to controls, ^###^*p* < 0.001, ^####^*p* < 0.0001 for differences compared to previous time point group.

To monitor the retinal morphological changes induced by a single intraperitoneal injection of MMS (75 mg/kg), retinal sections from multiple time points were stained by H&E. The normal mouse retinal structure was shown in Figure 1A. It was composed of retinal ganglion cell (RGC) layer, inner plexiform layer (IPL), inner nuclear layer (INL), outer plexiform layer (OPL), outer nuclear layer (ONL), inner segment and outer segment layer (IS/OS), and retinal pigment epithelium (RPE) from top to bottom. There were about 10–12 layers of neatly arranged photoreceptor cells located in the ONL. After MMS treatment, the thickness of the ONL and IS/OS decreased with the thickness of the ONL and IS/OS decreased with time (Figure 1B). One-way ANOVA revealed that the ONL became thinner with time after MMS injection [*F*_(8,72)_ = 862.1, *p* < 0.0001; Figure 1C]. *Post hoc* revealed that the thickness of ONL 4 and 12 h after MMS treatment was no difference to that of normal retina (*p* > 0.05). With the prolongation of MMS treatment, the ONL thickness gradually decreased, reaching the thinnest at 7 days (all *p* < 0.0001 at 1, 2, 3, 5, and 7 days). The thickness of IS/OS layer was also significantly reduced compared to the control group [*F*_(8,72)_ = 945.4, *p* < 0.0001; Figure 1D] and reached its thinnest at 7 days after MMS treatment (7 days vs. 5 days, *p* < 0.0001). The RPE layer was normal during the early stage, and only a few vacuolar changes were found at 5 and 7 days. There were no significant changes in the other retinal layers. The above results suggested that the photoreceptor cells were more sensitive to MMS than the other retinal cells. The cell degenerative changes appeared in a time-dependent manner.

### 3.2. Retinal structure was examined by optical coherence tomography

**Figure 2:**
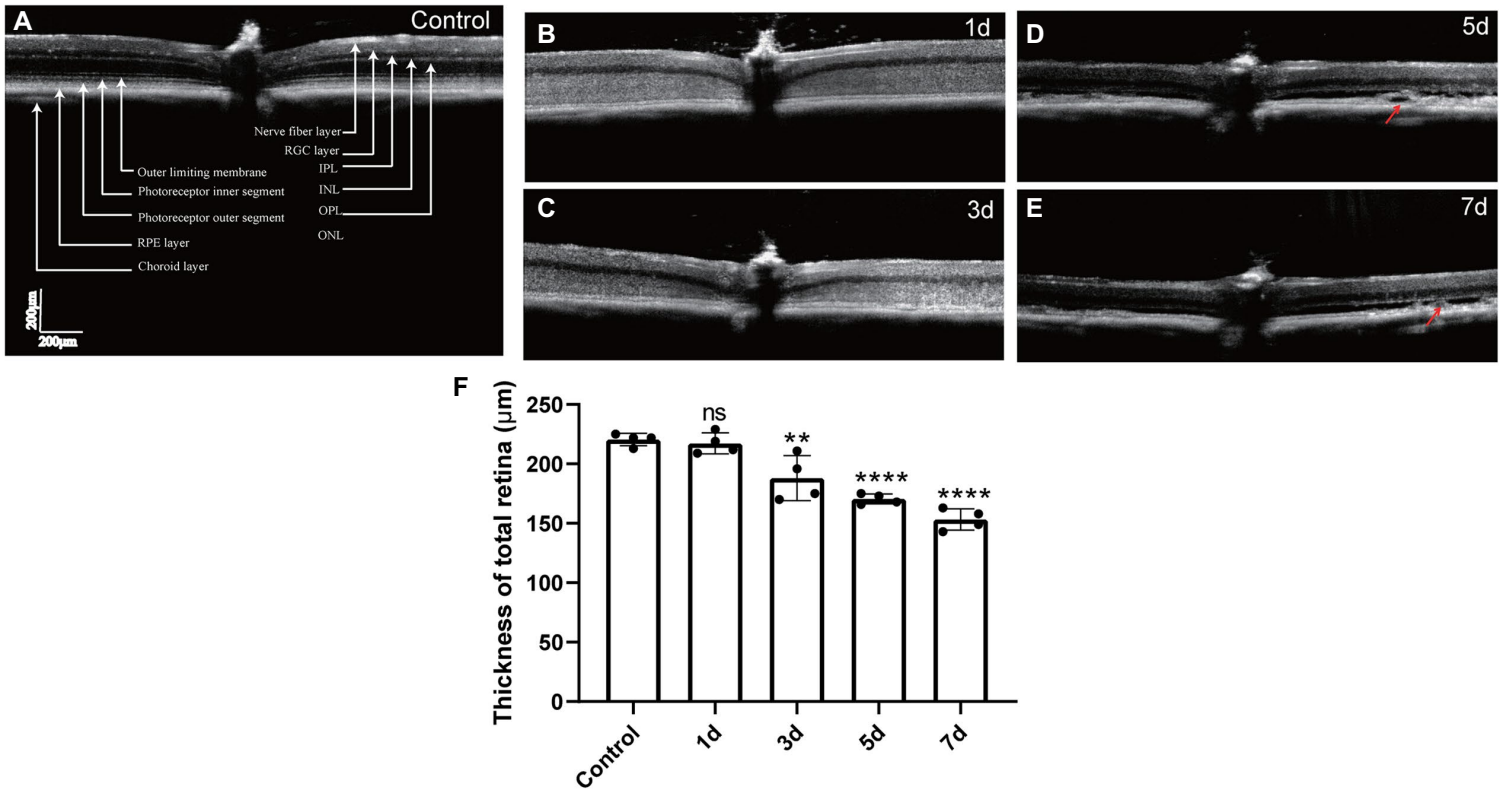
The representative OCT images of the retinal structure after MMS treatment with time. **(A)** the OCT images of the retinal layered structure in control mice. **(B–E)** The OCT images of control mice and MMS treated mice at different time points. The arrows showed the vacuoles occurred in the RPE layer at 5 and 7 days post MMS injection in panel **(D,E)**. (Scale bar, 200 μm) **(F)** The thickness of the total retina in the MMS treated group compared with the control group by one-way ANOVA, data were expressed as mean ± SD, *n* = 3/group, **p < 0.01, ****p < 0.0001 for differences compared to controls.

The retinal structures were also detected by non-invasive OCT *in vivo*. The retinal layers could be observed in the SD-OCT image of the mouse retina (Figure 2A). The density of ONL and IS/OS layers was hypo-reflective in control mice (Figure 2A), which became hyper-reflective from 1 to 3 days after MMS treatment. The border between the ONL and OPL was obscured and difficult to visualize (Figures 2B, C). However, the density turned back to hypo-reflective from 5 days post-injection, but the IS/OS was still difficult to distinguish. Consistent with the H&E staining images, local RPE showed swollen and vacuolization at 5 and 7 days (Figures 2D, E; arrows). One-way ANOVA analysis indicated that the thickness of the total retina decreased after MMS treatment with time [*F*_(4,15)_ = 30.11, *p* < 0.0001; Figure 2F]. The results suggested that the retinal structure changes could be detected *in vivo* by OCT as early as 24 h after MMS treatment. The hyper-reflective density of the ONL might represent the disarrangement of the ONL structure.

### 3.3. The retinal function was impaired progressively after MMS administration

**Figure 3:**
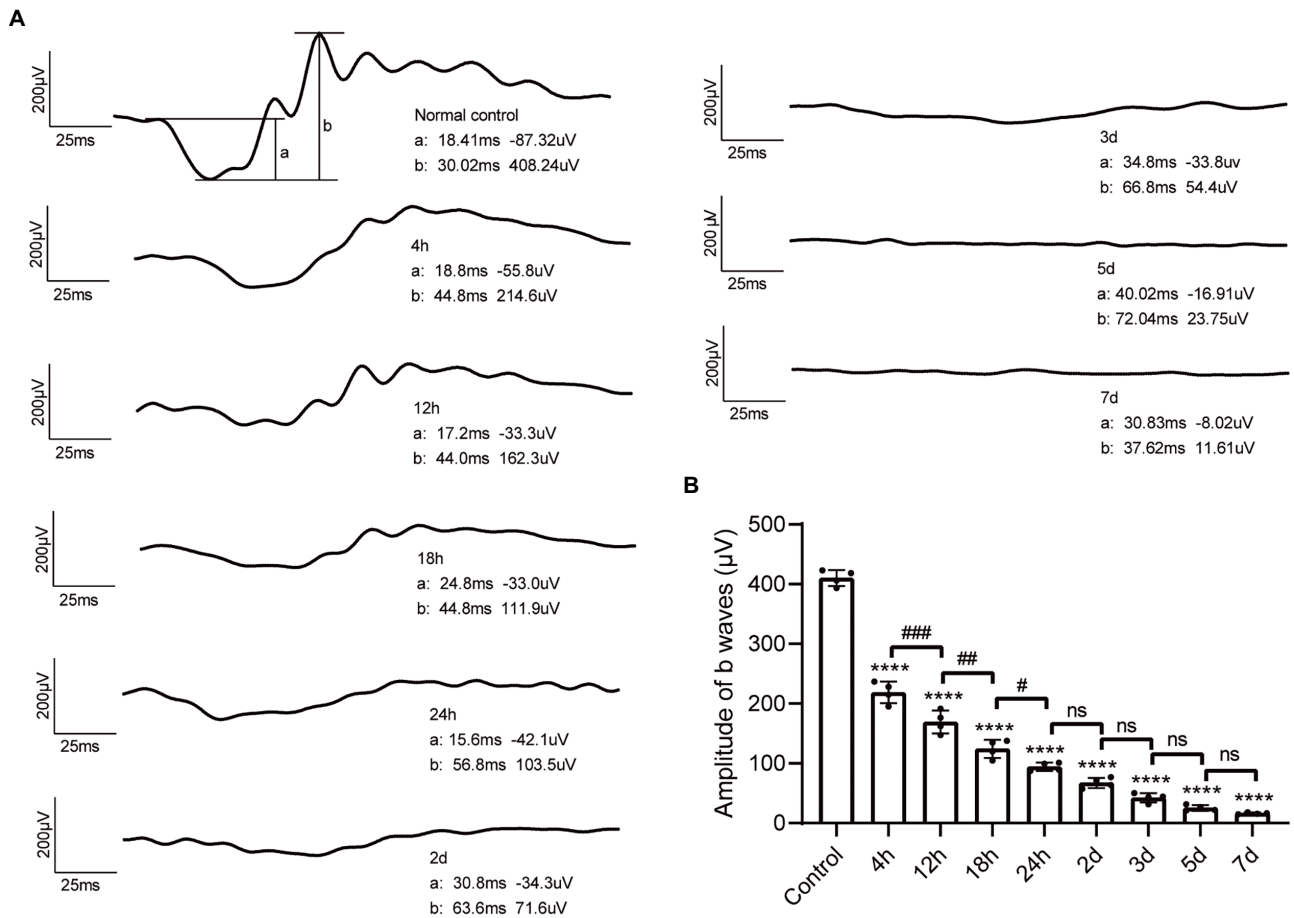
Changes of ffERG waveforms after MMS administration. **(A)** Representative waveform of dark-3.0 ERG recordings in control mice and MMS treated mice at different time points. **(B)** Quantification of b-wave amplitudes in the MMS treated mice compared to the controls. Data were expressed as mean ± SD, *n* = 4/group. One-way ANOVA analysis followed by Tukey’s *post hoc* test, ****p < 0.0001 for differences compared to controls, ^#^*p* < 0.05, ^##^*p* < 0.01, ^###^*p* < 0.001 for differences compared to the previous time point group.

Retinal function was evaluated by ffERG. Under the total dark-adapted condition, ERG waveforms were recorded according to the ISCEV Standard (McCulloch et al., 2015). The scotopic 3.0 ERG represents maximal combined responses arising from photoreceptors and bipolar cells, mainly from the rod system (McCulloch et al., 2015). Representative waveforms of control mice and MMS treated mice in different time points were shown in Figure 3A. Both a-wave and b-wave amplitudes decreased in MMS treated mice compared to the control. One-way ANOVA revealed that the a-wave and b-wave amplitudes were significantly lowered after MMS treatment with time [*F*_(8,27)_ = 438.3, *p* < 0.0001; Figure 3B]. The amplitudes of b-wave were not detected at 5 and 7 days.

### 3.4. Photoreceptor cell death detected by TUNEL assay after MMS treatment

**Figure 4:**
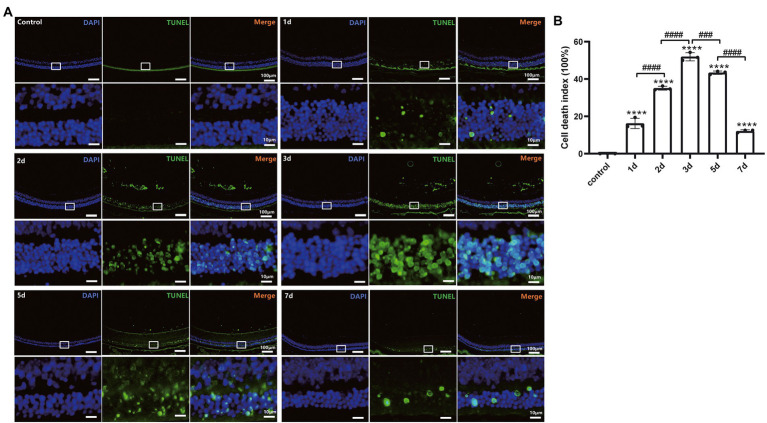
MMS-induced photoreceptor cell death labeled by TUNEL. **(A)** Representative retinal image for TUNEL and DAPI co-staining of control and MMS treated mice at different time points. 10×, Scale bar = 100 μm; The zoom-in images were posted right below the originals with 10 times magnification. Scale bar = 10 μm. **(B)** Quantification of cell death index in retinal sections after MMS administration at different time points. Data were expressed as mean ± SD, *n* = 3/group. One-way ANOVA analysis followed by Tukey’s *post hoc* test, ****p < 0.0001 for differences compared to controls, ^###^*p* < 0.001, ^####^*p* < 0.0001 for differences compared to previous time point group.

The photoreceptor cell death induced by MMS treatment was estimated with TUNEL assay. As shown in Figure 4A, the TUNEL-positive cells were mainly located in the ONL. One-way ANOVA revealed that the cell death index (CDI) significantly increased after MMS administration compared to the controls and peaked at 3 days and then slightly decreased at 5 days [*F*_(5,12)_ = 494.7, *p* < 0.0001; Figure 4B]. Only a few TUNEL positive cells were detected at 7 days. These findings demonstrated MMS induced retinal damage targeted photoreceptor cells specifically.

### 3.5. DNA methylation associated regulators were changed during MMS-induced retinal damage

**Figure 5:**
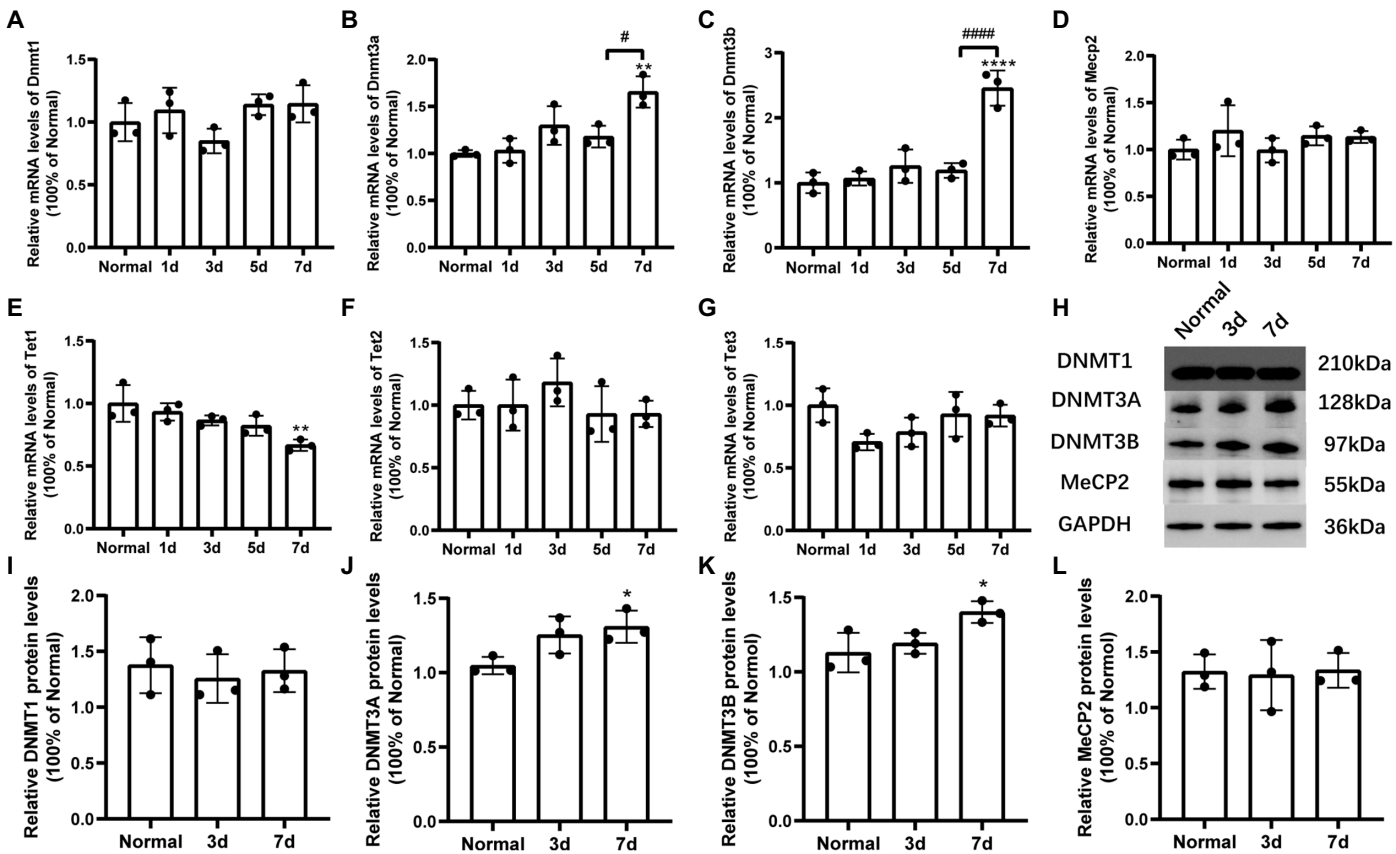
Changes of mRNA and protein levels of DNA methylation-related factors induced by MMS treatment. **(A–G)** The mRNA levels of *Dnmt1/3a/3b*, *Mecp2*, and *Tet1/2/3* at 7 days after MMS treatment compared to Normal groups. **(H)** Representative immunoblots of DNMT1/3A/3B, MeCP2, GAPDH were used as an internal control. **(I–L)** Quantitative analysis of protein levels related to GAPDH. Data were expressed as mean ± SD, *n* = 3/group. *p < 0.05, **p < 0.01, ****p < 0.0001 for differences compared to Normal groups, ^#^*p* < 0.05, ^####^*p* < 0.0001 for differences compared to previous time point group.

The expression levels of DNA methylation and DNA demethylation associated regulators in retina were determined by real-time qPCR (Figures 5A–G) and western blot techniques (Figures 5H–L). The mRNA levels of Dnmt3a/3b were upregulated significantly at 7 days after MMS treatment compared to the control group [*F*_Dnmt3a(4,10)_ = 10.30, *p* = 0.0014; Figure 5B; *F*_Dnmt3b(4,10)_ = 28.92, *p* < 0.0001; Figure 5C], and the relative protein levels of DNMT3A/3B were also increased correspondingly [t_DNMT3A-7d_ = 3.693, *p* = 0.0210; Figure 5J; t_DNMT3B-7d_ = 3.112, *p* = 0.0358; Figure 5K]. However, there was no changes in expression of DNMT1 and MeCP2 (*p* > 0.05, Figures 5A, D, I, L). Moreover, DNA demethylation associated enzymes (Tet1/2/3) were also detected using RT-qPCR. Tet1 was downregulated at 7 days [*F*_Tet1(4,10)_ = 6.578, *p* = 0.0073; Figure 5E], while Tet2 and Tet3 did not shown significant differences (*p* > 0.05; Figures 5F, G).

### 3.6. 5-aza-dC ameliorated retinal morphology and function of MMS treated mice

An *in vitro* report showed that treatment with DNMT inhibitor 5-aza-dC could delay photoreceptor degeneration in rd1 retinal explants (Farinelli et al., 2014). Therefore, we applied 5-aza-dC (2μM, 1μL) by intravitreal injection one day before MMS administration to investigate whether 5-aza-dC has any effect on photoreceptor survival *in vivo*. After MMS treatment for 7d, the retinal morphology was observed by H&E staining.

**Figure 6:**
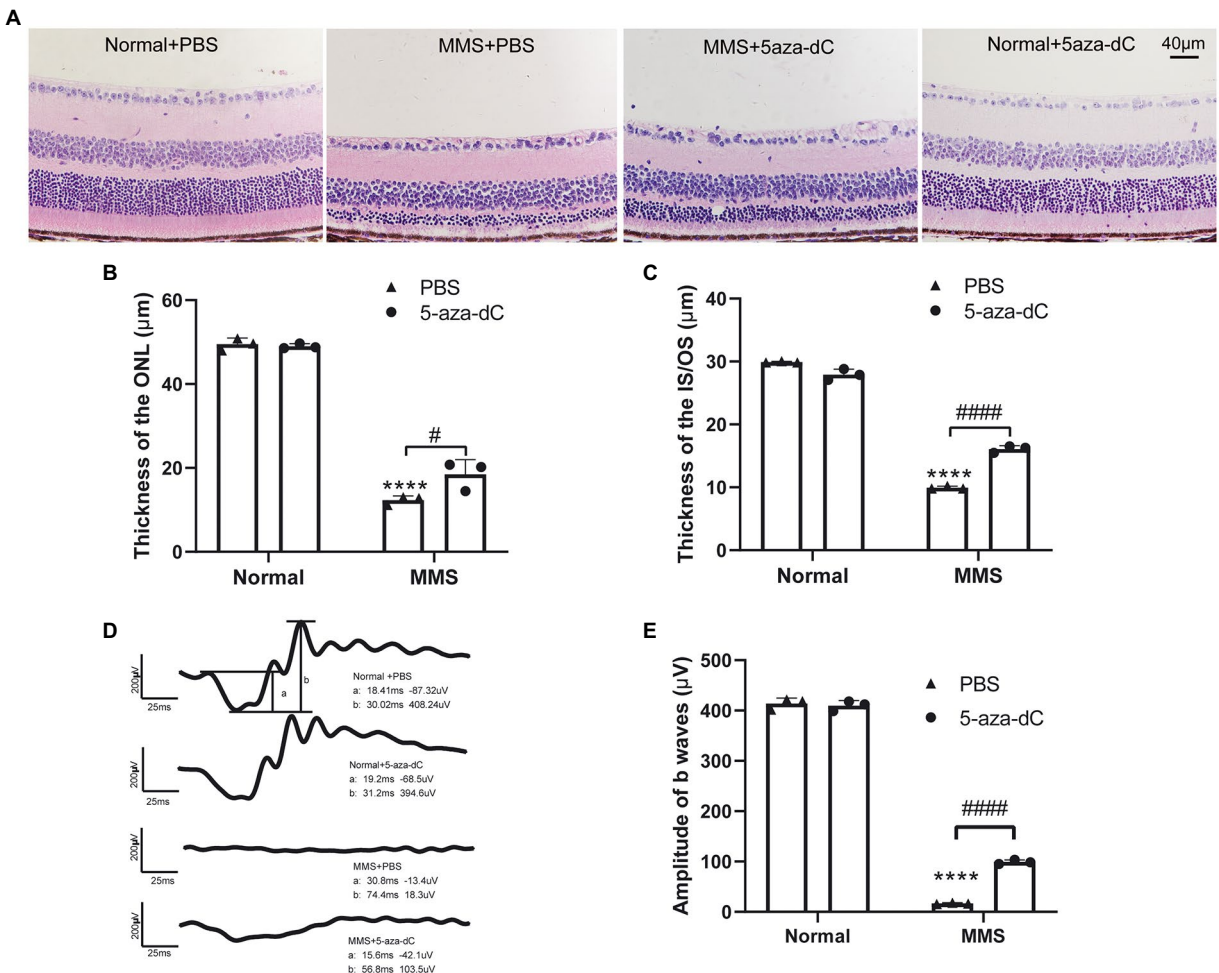
5-aza-dC attenuated the toxic effects of MMS treatment. **(A)** Representative H&E staining images of retina at 7 days after MMS treated mice with or without 5-aza-dC. Scale bar, 40 μm. **(B,C)** The thickness of the ONL and IS/OS in the MMS + 5-aza-dC group was significantly increased than that in the MMS + PBS group. **(D)** Representative scotopic 3.0 ERG waveforms at 7 days after MMS treated mice with or without 5-aza-dC, scale bar: 200 μV, 25 ms. **(E)** The amplitudes of b waves in the MMS + 5-aza-dC group was significantly increased than that in the MMS + PBS group. Data were expressed as mean ± SD, *n* = 3/group. Two-way ANOVA followed by Sidak’s *post hoc* test, ****p < 0.0001 for differences compared to Normal+PBS group, ^#^*p* < 0.05, ^####^*p* < 0.0001 for differences between groups.

The retinal morphology was observed by H&E staining. As indicated in the representative images (Figure 6A), there were obvious differences in the thickness of ONL and IS/OS layer in the MMS treatment group compared to the control group. In the MMS + 5-aza-dC group, the structure of the IS/OS was partially alleviated compared to the MMS + vehicle group, and the connecting cilium between inner segment and outer segment was clearly visible. Two-way ANOVA analysis revealed that both MMS and 5-aza-dC had effect on the thickness of the ONL (Figure 6B) and IS/OS (Figure 6C) [*F*_ONL-MMS(1,8)_ = 884.9, *p* < 0.0001; *F*_ONL-5aza(1,8)_ = 6.146, *p* = 0.0382; *F*_IS/OS-MMS(1,8)_ = 2,760, *p* < 0.0001; *F*_IS/OS-5aza(1,8)_ = 1.592, *p* = 0.0001]. There was significant interactive effect between MMS and 5-aza-dC [*F*_ONL-MMS × 5-aza-dC(1,8)_ = 8.616, *p* = 0.0188; *F*_IS/OS-MMS × 5-aza-dC(1,8)_ = 180.2, *p* < 0.0001]. These results demonstrated that the photoreceptor cell layers were damaged by MMS at 7 days, and 5-aza-dC significantly attenuated the toxic effects of MMS on the thickness of the ONL.

The retinal function was evaluated by ffERG. Representative images of scotopic-3.0 ERG responses were shown in Figure 6D. Two-way ANOVA revealed that both MMS and 5-aza-dC have an effect on the b-wave of dark-3.0 ERG [*F*_MMS(1,8)_ = 6,657, *p* < 0.0001; *F*_5-aza-dC(1,8)_ = 80.96, *p* < 0.0001; *F*_MMS × 5-aza-dC(1,8)_ = 99.53, *p* < 0.0001; Figure 6E]. The results demonstrated that 5-aza-dC significantly improved the retinal function of MMS treated mice, which was consistent with the retinal morphological changes.

### 3.7. 5-aza-dC decreased the protein levels of DNA methylation associated factors

**Figure 7:**
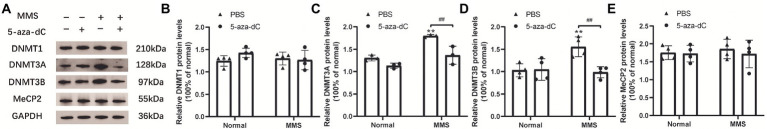
The interactive effect on protein levels of DNA methylation-related factors between MMS and 5-aza-dC. **(A)** Representative immunoblotting images of DNMT1/3A/3B, MeCP2 at 7 days after MMS treatment, GAPDH was used as an internal control. **(B–E)** Quantitative analysis of protein levels related to GAPDH. Data were expressed as mean ± SD, *n* = 3–4/group. **p < 0.01 for differences compared to controls, ^##^*p* < 0.01 for differences between groups.

The whole retina in each group were extracted at 7 days after MMS injection. And the protein levels of DNMT1/3A/3B and MeCP2 were evaluated by western blot technique. The representative immunoblotting images were shown in Figure 7A. The quantitative expression levels of DNMT3A [*F*_MMS(1,8)_ = 32.39, *p* = 0.0005; *F*_5-aza-dC(1,8)_ = 22.58, *p* = 0.0014; *F*_MMS × 5-aza-dC(1,8)_ = 4.011, *p* = 0.0802; Figure 7C] and DNMT3B [*F*_MMS(1,12)_ = 5.929, *p* = 0.0314; *F*_5-aza-dC(1,12)_ = 8.380, *p* = 0.0135; *F*_MMS × 5-aza-dC(1,12)_ = 9.546, *p* = 0.0094; Figure 7D] were significantly influenced by MMS and 5-aza-dC. While there were no changes in DNMT1 and MeCP2 (Figures 7B, E; *p* > 0.05). These results suggested that the MMS could increase the protein levels of DNMT3A/3B, whereas the 5-aza-dC, as a DNMT inhibitor, had an inverse effect to downregulate the expression levels, which hinted that 5-aza-dC might attenuate the retinal structure and function *via* changing the DNA methyltransferases.

## 4. Discussion

Retinitis pigmentosa (RP) is one of the most common inherited retinal degenerative diseases in clinical practice. Numerous investigations were performed to identify the underlying pathological mechanisms of RP and explore safer and more effective therapeutic strategies that are easier to implement. A good animal model is essential for the investigation. Multiple animal models have been used in RP researches, including gene mutation animals (rd1, rd10 mice, RCS, P23H, S334ter rats, et al.; Kaur et al., 2011, Sahaboglu et al., 2020, Napoli et al., 2021, He et al., 2022), chemical-induced retinal degeneration (MMS, MNU, and NaIO3; Allocca et al., 2019, Tao et al., 2019a, Wang et al., 2022), and light-induced photoreceptor cell damage (Napoli and Strettoi, 2022). The common mechanisms of cell death in RP include inflammation, apoptosis, necrosis, and autophagy (Olivares-Gonzalez et al., 2021). Among them, the parthanatos is a new form of regulated necrosis that depends on the overexpression of PARP. The PARP hyperactivation-related cell death mechanism was detected in several RP models, such as rd1 and rd10 mice (Pde6β mutation), P23H, and S334ter rats (RHO mutation), as well as in AMD model that exposed to oxidative stress (Kaur et al., 2011; Jang et al., 2017; Olivares-González et al., 2020; Sahaboglu et al., 2020). Previous evidence showed that the PARP1 hyperactivation-related cell death also existed in MMS-induced photoreceptor cell death models. DNA alkylating damages (7meG, 3meA) trigger base excision repair (BER), which is initiated by alkyladenine DNA glycosylase (AAG; Calvo et al., 2013; Allocca et al., 2019). During the BER process, PARP1 is activated and bound to the DNA single-strand breaks (SSBs), and recruits the XRCC1-polβ-DNA ligase III complex to complete DNA repair. However, if unrepaired, hyperactivating PAPR1 catalyzes the formation of PAR polymers with NAD^+^ as a substrate, causing substrate depletion, bioenergetic failure, and cell death. Moreover, the PAR polymers can be transferred to the mitochondria which promote apoptosis-inducing factor (AIF) to translocate to the nucleus, causing DNA degradation and cell death. In addition, the PAR polymers could restrain glycolysis by binding and inhibiting hexokinase in mitochondria, leading to cell death (Andrabi et al., 2014). Therefore, the alkylating agents induced RP animal models have the same metabolic toxicity as in different mutation models. The common mechanisms of cell death provide new therapeutic targets independent of any mutations.

In the present study, we administered MMS (75 mg/kg) by intraperitoneal injection to induce selective retinal photoreceptor degeneration. The thickness of the ONL was the thinnest at 7 days after MMS treatment. The dying cells were located in the ONL, which indicated that MMS mainly induced photoreceptor cell death without influencing the other retinal neurons. As mentioned above, AAG, the initiator of BER (Meira et al., 2009), is not only located in the nucleus but also detected in the mitochondria (van Loon and Samson, 2013). Retinal photoreceptors are light-sensitive neurons that demand an extreme energy metabolism to maintain the phototransduction and renew the discs of the outer segments. The density of mitochondria in photoreceptors is much higher than that in any other cells in the body (Hoang et al., 2002; Fu et al., 2019). This may explain some of the reasons why the photoreceptors are the primary affected neurons by MMS treatment.

Though the number of mutation genes linked to RP has exceeded 90^1^, in many cases there is no relationship between them, which suggests that a novel process may be associated with the pathogenesis of the photoreceptor degeneration. DNA methylation and demethylation are important epigenetic mechanisms in the pathogenesis of retinal diseases. Based on this, Dvoriantchikova et al. (2022) proposed an epigenetic RP model by suppressing demethylation of genes which are essential for transition from RPC-to-photoreceptor without any mutations. DNA methyltransferase and demethylase have been recognized to be involved in the repair of C/T mismatch caused by deamination of methylated cytosine in BER pathway, and PARP1 could regulate the activity of target protein through recruitment of demethylases (Maiti and Drohat, 2011; Weber et al., 2016; Sutcu et al., 2019; Jiang et al., 2020). Oxidative stress and oxidation-dependent DNA damage are major risk factors in retinal degeneration-associated photoreceptor cell death (Komeima et al., 2006; Huang et al., 2018), and MMS-induced DNA damage and BER are actively implicated in the modulation of DNA methylation and demethylation (Jiang et al., 2020). 7MeG and 3MeA, produced by the reaction of MMS and oxygen stress, might be demethylated by inhibiting the activity of DNMTs, leads to base loss and nucleotide misincorporation during BER (Lutsenko and Bhagwat, 1999; Rošić et al., 2018). Furthermore, in the course of BER, DNA polymerase β (pol β) interacts with DNMT3B to recruit DNMTs to the region proximate to the oxidized DNA bases, resulting in *de novo* DNA methylation at the adjacent CpGs (Lai et al., 2016; Jiang et al., 2020).

Here, we demonstrated that the retinal photoreceptor degeneration process induced by MMS was accompanied by increased expression of *Dnmt3a/3b* and decreased expression of DNA demethylase *Tet1*. The variation of DNMTs may be triggered by MMS stimulus (Moore et al., 2013), or may be recruited by mismatched repair proteins or pol β during the BER process causing changes in DNA methylation patterns (Ding et al., 2016). Recent studies have also shown that DNA oxidative damage interacts with histone modifiers including SIRT1 and EZH2 to regulate GC-rich regions of the genome, regulating gene methylation and transcriptional activity (O’Hagan et al., 2011; Maiuri et al., 2017). DNA methylation and gene regulation are involved in photoreceptor cell death. Farinelli et al. (2014) found the mRNA levels of DNMT3A and 3 L were upregulated in the rd1 retina and cytosine methylation was increased at the peak of degeneration. Farinelli et al. (2014) also made a point about DNA methylation being a late event in photoreceptor cell death. Consistent with this, there has been evidence for changes in the levels of DNA methylation-associated regulators at the late stage of MMS induced retinal damage in the present study, which could support this viewpoint as well. Aberrant DNA methylation may not be the initiator of the diseases, but may be associated with the progression of diseases (Schoofs et al., 2013).

Decitabine (5-aza-dC), a nucleoside analog, acts as a suicide inhibitor of DNA methyltransferase when covertly incorporated into DNA (Zhou et al., 2018). 5-aza-dC allows transcription factors to bind to promoter regions, assembly of transcription complexes, and subsequent gene expression by loosening chromatin structure (Flagiello et al., 2002). Treatment with 5-aza-dC restored the gene expression of *SOD2* and *GPx2* in cells challenged with lipid peroxidation (Yara et al., 2013). An *in vitro* study of rd1 mice showed that the application of 5-aza-dC to the retinal explant cultures could decrease DNA methylation level and rescue the retinal photoreceptor degeneration in the short-term. While there is no effect in the long-term (Farinelli et al., 2014). In this study, 5-aza-dC was applied to the eye before MMS treatment. It was found that acute damage to photoreceptor cells induced by MMS was significantly alleviated. Although the photoreceptor cell layer of mice in MMS + 5-aza-dC treated group was still aberrant, the ONL and IS/OS layers were thicker than that of the MMS + vehicle group within 7 days, and the retinal function was partially restored. These results demonstrated that there was protective effect of 5-aza-dC on acute retinal damage. The long-term effect of decitabine treatment in this RP model needs further studies. In addition to the positive effect of DNA methyltransferase inhibition, 5-aza-dC has been demonstrated toxicity and potential mutagenicity. It can also induce a DNA-repair response (Rogstad et al., 2009). Therefore, it is necessary to determine the optimal dosage of 5-aza-dC. Consistent with previous findings (Chen et al., 2020), our preliminary results showed that 5-aza-dC (2 μM, 1 μl) intravitreal injection has no effect on the body weight and locomotor activity of mice (not shown in the figure), and does not induce photoreceptor cell death and retinal function impairment (Figure 6). However, the optimal dose schedule for 5-aza-dC with a novel mechanism of action remains to be determined (Kantarjian and Issa, 2005).

In conclusion, our results demonstrated that intraperitoneal injection of 75 mg/kg MMS specifically induced retinal photoreceptor cell death in mice, which was accompanied by the changes in regulatory factors of DNA methylation. 5-aza-dC delayed MMS-induced photoreceptor cell death and partially restored retinal function, probably by inhibition of DNMT3A and DNMT3B. Thus, the appropriate dose of DNMT inhibitors may have therapeutic potential for chemical-induced retinal degeneration. Further studies are required to fully understand the mechanisms of DNA methylation in retinal degeneration-related diseases.

## Data availability statement

The original contributions presented in the study are included in the article/Supplementary material, further inquiries can be directed to the corresponding author.

## Ethics statement

The animal study was reviewed and approved by the Animal Research Ethics Committee of Zhengzhou University.

## Author contributions

G-HP and YJ designed all experiments. YJ and MZ performed the experiments. YJ, XQ, and MZ analyzed the data and wrote the manuscript. All authors commented on this manuscript. G-HP provided support and supervised the project. All authors contributed to the article and approved the submitted version.

## Funding

This research is supported by grants from the National Key Research and Development Program (2018YFA0107303) and the Natural Science Foundation of China (82070990).

## Conflict of interest

The authors declare that the research was conducted in the absence of any commercial or financial relationships that could be construed as a potential conflict of interest.

## Publisher’s note

All claims expressed in this article are solely those of the authors and do not necessarily represent those of their affiliated organizations, or those of the publisher, the editors and the reviewers. Any product that may be evaluated in this article, or claim that may be made by its manufacturer, is not guaranteed or endorsed by the publisher.

## Footnotes

^1^ https://web.sph.uth.edu/RetNet/
