# Supplementary figures and images for: Decitabine improves MMS-induced retinal photoreceptor cell damage by targeting DNMT3A and DNMT3B
Source: Front Mol Neurosci. 2023 Jan 10;15:1057365. doi: 10.3389/fnmol.2022.1057365 (PMC9872157; doi:10.3389/fnmol.2022.1057365)

DNMT3B

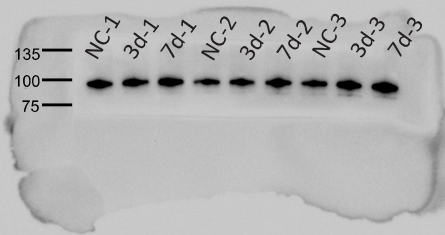

DNMT3A

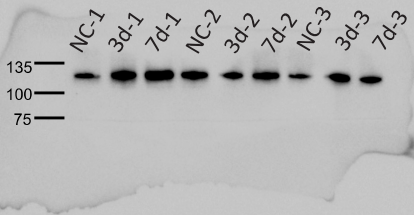

MeCP2

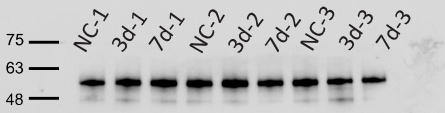

GAPDH

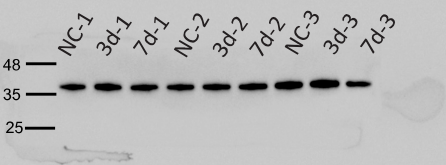

DNMT1-1

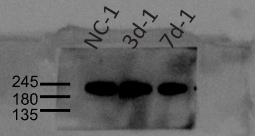

DNMT1-2

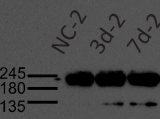

DNMT1-3

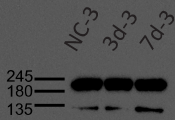

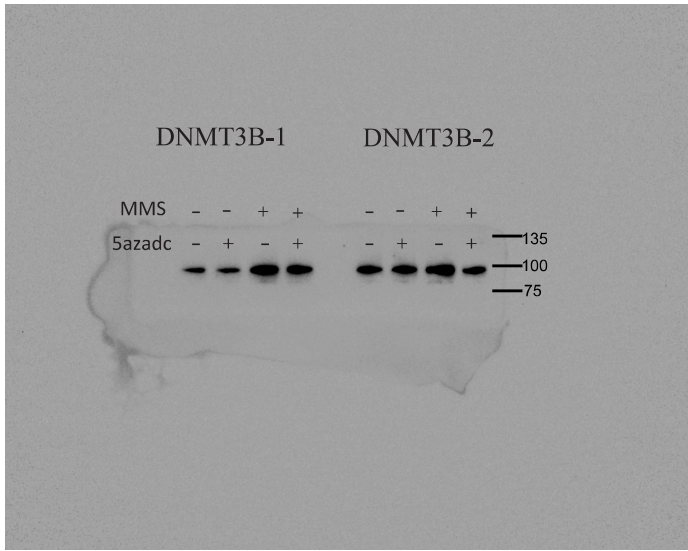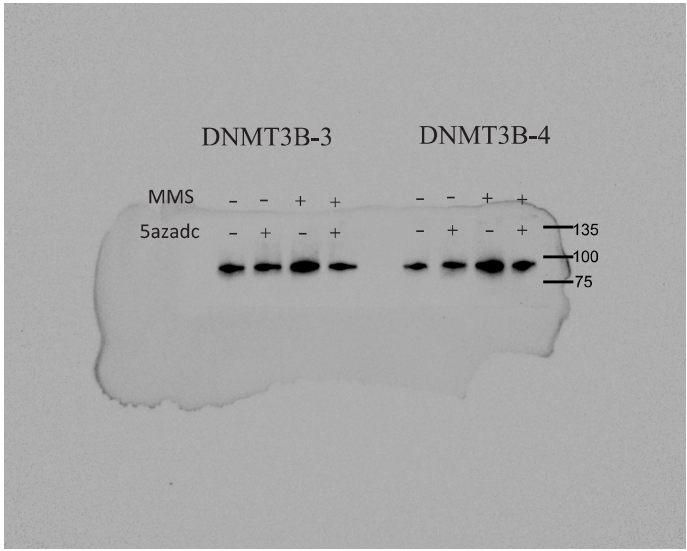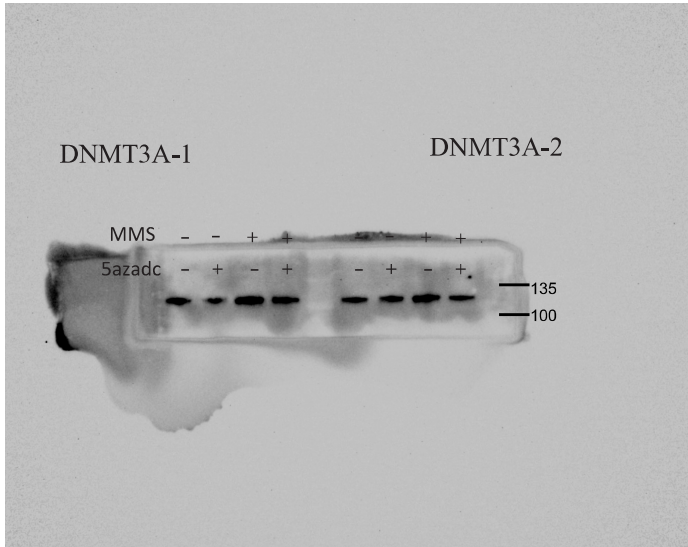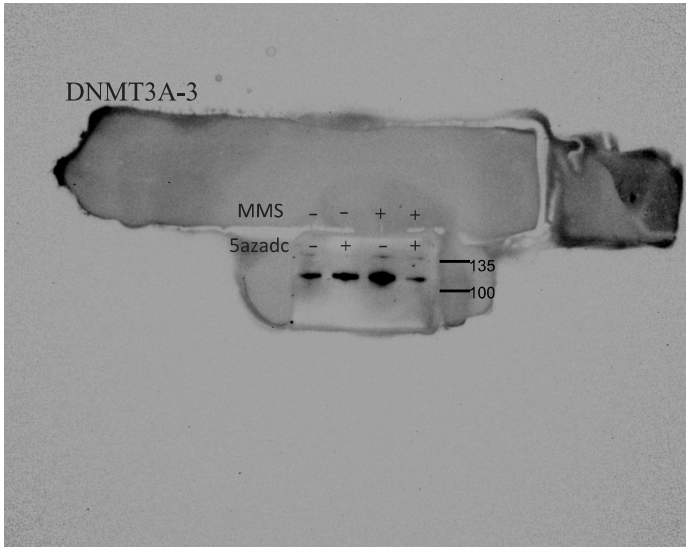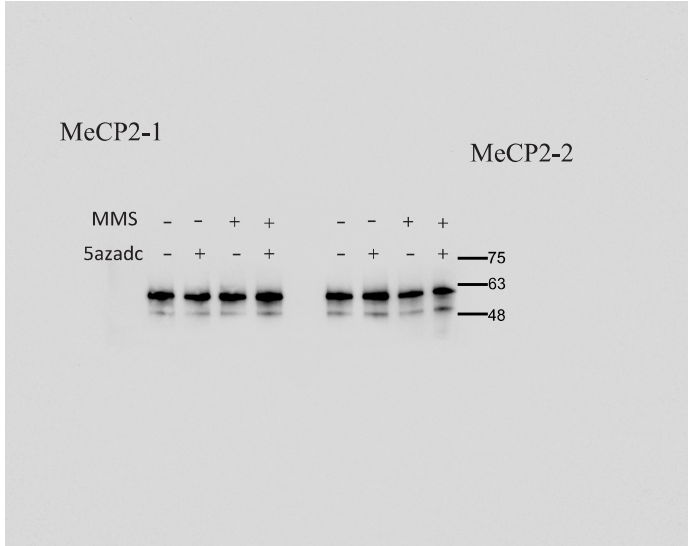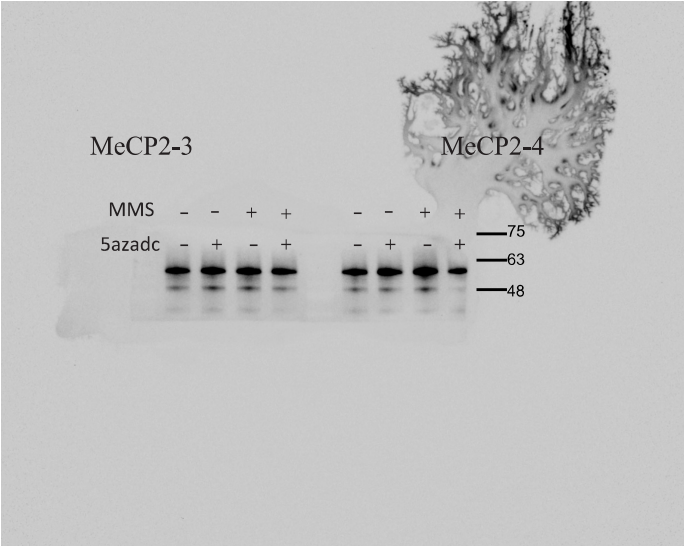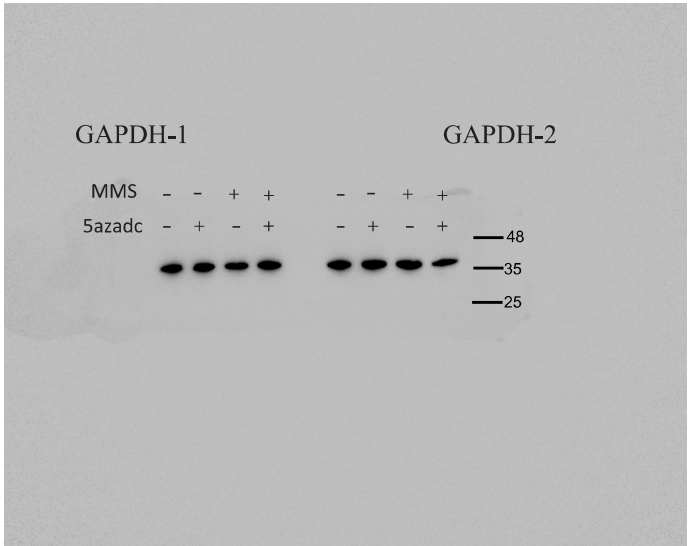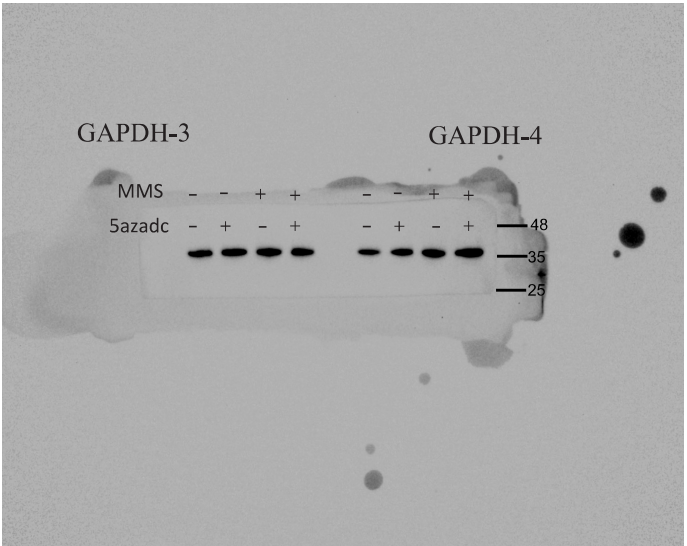

DNMT1-1

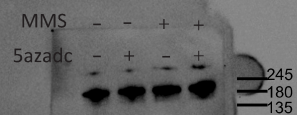

DNMT1-2

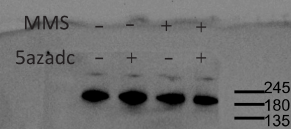

GAPDH-1

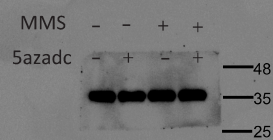

GAPDH-2

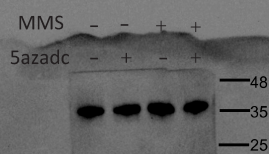

DNMT1-3

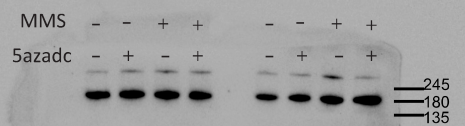

DNMT1-4

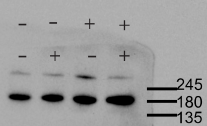

GAPDH-3

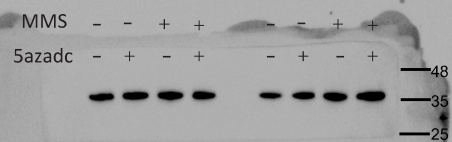

GAPDH-4

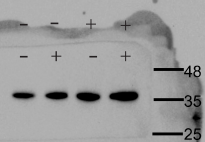

Supplement: Supplementary file 1 [file Data_Sheet_1.PDF]
